# Supplementary material for: Modulation of the Immune Response by Deferasirox in Myelodysplastic Syndrome Patients
Source: Pharmaceuticals (Basel). 2021 Jan 7;14(1):41. doi: 10.3390/ph14010041 (PMC7825690; doi:10.3390/ph14010041)
Supplement: Supplementary file 1 [file pharmaceuticals-14-00041-s001.zip › Supplement files/Table S2.docx]

| GO ID | Biological process | Count | P-value | Genes |
| --- | --- | --- | --- | --- |
| GO:0007049 | cell cycle | 6 | 1,60E-02 | E2F2, RASSF2, SMARCB1, CCPG1, HMG208, TP53 |
| GO:0002250 | adaptive immune response | 5 | 1,80E-02 | CD79B, CTSH, DBNL, PIK3CD, PAG1 |
| GO:0006338 | chromatin remodeling | 4 | 2,10E-02 | SMARCB1, SMARCD2, BAZ1A, MSL3 |
| GO:0097368 | establishment of Sertoli cell barrier | 2 | 2,70E-02 | ARID4B, RAB13 |
| GO:0045876 | positive regulation of sister chromatid cohesion | 2 | 2,70E-02 | RAD21, FEN1 |
| GO:0031175 | neuron projection development | 4 | 3,10E-02 | RAB13, UHMK1, CAPZB, PRMT1 |
| GO:0006457 | protein folding | 5 | 3,40E-02 | FKBP2, CSNK2A1, ERP29, CANAB, PDIA6 |
| GO:0032755 | positive regulation of interleukin-6 production | 3 | 3,70E-02 | TBC1D23, ADORA2B, ZBTB20 |
| GO:0098609 | cell-cell adhesion | 6 | 3,70E-02 | CAPZB, DBN1, DBNL, IDH1, PAK2, PFN1 |
| GO:0016575 | histone deacetylation | 3 | 3,90E-02 | ARID4B, HMG20B, MSL3 |
| GO:0006915 | apoptotic process | 9 | 3,90E-02 | BCAP29, RAD21, BIRC3, CSNK2A1, CTSH, CYFIP2, FOXO1, PAK2, TP53 |
| GO:0006351 | transcription, DNA-templated | 21 | 4,00E-02 | ARID4B,DDX17,NME1-NME2, RALY,SMARCB1, SMARCD2, BAZ1A,CSNK2A1,DPPA4, FOXO1,HMG20B,IRF8,MSL3,NFIC,NFE2L3,NCOA5,TP53,ZBTB20, ZNF277,ZNF302,ZNF669 |

| KEGG ID | Pathway | | Count | | P-value | | Genes | |
| --- | --- | --- | --- | --- | --- | --- | --- | --- |
| hsa05220 | Chronic myeloid leukemia | | 4 | | 1,60E-02 | | CRK, E2F, PIK3CD, TP53 | |
| hsa04810 | Regulation of actin cytoskeleton | | 6 | | 1,90E-02 | | CRK, CYFIP2, PAK2, PIK3CD, PIP5K1B, PFN1 | |
| hsa05222 | Small cell lung cancer | | 4 | | 2,50E-02 | | E2F, BIRC3, PIK3CD, TP53 | |
| hsa05215 | Prostate cancer | | 4 | | 2,70E-02 | | E2F, FOXO1, PIK3CD, TP53 | |
|  |  |  | |  | |  | |  |
