# Supplementary material for: Modulation of the Immune Response by Deferasirox in Myelodysplastic Syndrome Patients
Source: Pharmaceuticals (Basel). 2021 Jan 7;14(1):41. doi: 10.3390/ph14010041 (PMC7825690; doi:10.3390/ph14010041)
Supplement: Supplementary file 1 [file pharmaceuticals-14-00041-s001.zip › Supplement files/Table S5.docx]

| GO ID | Biological process | Count | P-value | Genes |
| --- | --- | --- | --- | --- |
| GO:0050832 | defense response to fungus | 5 | 3,60E-06 | S100A8, S100A9, DEAF1, DEFA1B, DEFA3 |
| GO:0002227 | innate immune response in mucosa | 4 | 1,30E-04 | CAMP, DEAF1, DEFA1B, DEFA3 |
| GO:0006955 | immune response | 9 | 2,40E-04 | CTSW, DEAF1, DEFA1B, IRF8, IL1B, LTB, HLA-DMB, OSM |
| GO:0045087 | innate immune response | 9 | 2,70E-04 | S100A8, S100A9, CAMP, CYBB, DEAF1, DEFA1B, DEFA3, HMGB3, LY86 |
| GO:0019731 | antibacterial humoral response | 4 | 6,80E-04 | CAMP, DEAF1, DEFA1B, DEFA3 |
| GO:0031640 | killing of cells of other organism | 3 | 1,30E-03 | DEAF1, DEFA1B, DEFA3 |
| GO:0045638 | negative regulation of myeloid cell differentiation | 3 | 2,50E-03 | MEIS1, HMGB3, ZBTB16 |
| GO:0042742 | defense response to bacterium | 5 | 2,50E-03 | S100A8, S100A9, CAMP, DEFA3, IRF8 |
| GO:0050729 | positive regulation of inflammatory response | 4 | 3,00E-03 | S100A8, S100A9, FABP4, PLA2G7 |
| GO:0030520 | intracellular estrogen receptor signaling pathway | 3 | 3,00E-03 | DEAF1, DEFA1B, DEFA3 |
| GO:0001816 | cytokine production | 3 | 4,30E-03 | S100A8, S100A9, FABP4 |
| GO:0050830 | defense response to Gram-positive bacterium | 4 | 4,60E-03 | CAMP, DEAF1, DEFA1B, DEFA3 |
| GO:0070488 | neutrophil aggregation | 2 | 7,80E-03 | S100A8, S100A9 |
| GO:0002540 | leukotriene production involved in inflammatory response | 2 | 7,80E-03 | ALOX5AP, ALOX5 |

| KEGG ID | | Pathway | Count | | P-value | | Genes | |
| --- | --- | --- | --- | --- | --- | --- | --- | --- |
| hsa05152 | | Tuberculosis | 5 | | 1,60E-02 | | RAB5C, CAMP, IL1B, HLA-DMB, STAT1 | |
| hsa04060 | | Cytokine-cytokine receptor interaction | 5 | | 4,40E-02 | | TNFRSF21, IL1B, IL11RA, LTB, OSM | |
| hsa05321 | | Inflammatory bowel disease (IBD) | 3 | | 4,80E-02 | | IL1B, HLA-DMB, STAT1 | |
|  |  | |  |  | |  | |  |
